# Supplementary material for: Stress-induced ribosome degradation in Bacillus subtilis is mediated by the RNase Y-specificity complex
Source: Nat Commun. 2026 Jun 2;17:4886. doi: 10.1038/s41467-026-73310-x (PMC13230545; doi:10.1038/s41467-026-73310-x)
Supplement: Supplementary file 1 — Supplementary Information [file 41467_2026_73310_MOESM1_ESM.pdf]

Supplementary Information

Stress-induced ribosome degradation in *Bacillus subtilis* is mediated by the RNase Y-specificity complex

Fabián A. Cornejo[1,#,*], Kristina Driller[1,2,#], Rina Ahmed-Begrich[1], Katja Schmidt[1], Michael Jahn[1], Vivekanandan Shanmuganathan[1], Karin Hahnke[1], Florian Kondrot[1], Thomas F. Wulff[1], Sebastian Rämisch[1], Kathirvel Alagesan[1], Emmanuelle Charpentier[1,3], and Kürşad Turgay[1,2,*]

[1]Max Planck Unit for the Science of Pathogens, Berlin, Germany

[2]Leibniz Universität Hannover, Institute of Microbiology, Hannover, Germany

[3]Humboldt-Universität zu Berlin, Institute of Biology, Berlin, Germany

[#]These authors equally contributed to this work

**Correspondence to*: cornejo@mpusp.mpg.de, turgay@mpusp.mpg.de

Supplementary Figures

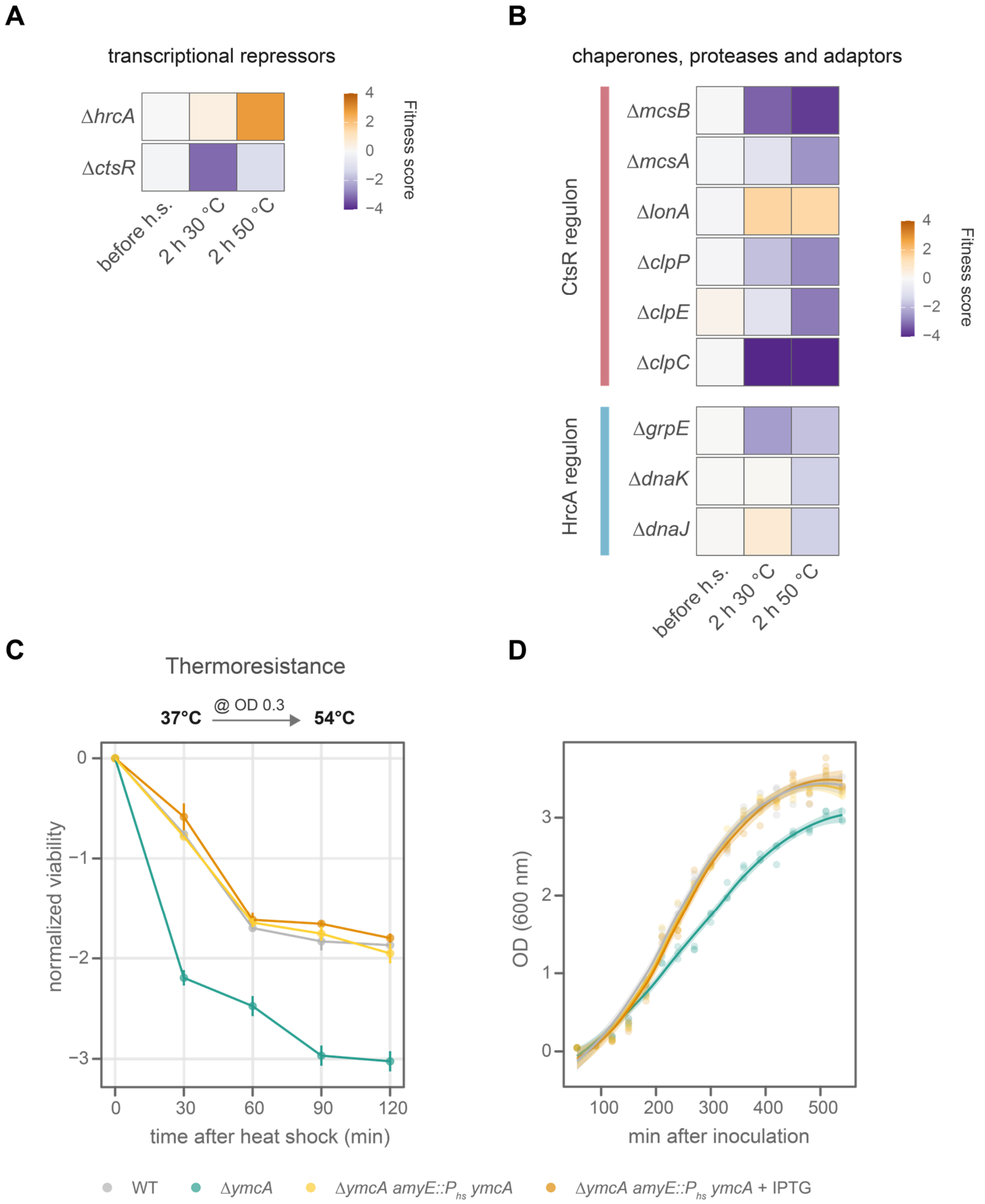

Supplementary Figure 1. Validation of the genetic screening approach and Y-complex-dependent phenotypes. Heatmap of the relative fitness of **A)** heat shock transcriptional repressors, and **B)** chaperones, proteases and adaptors. The color represents the average relative fitness of three biological replicates. A negative relative fitness value means that the abundance of the deletion mutant, compared to the rest of the population, was negatively affected by the treatment, while a positive value means an increased abundance on those conditions. h.s. = heat shock. **C)** Changes in the viability (log_{10}CFU/ml) of WT (grey) and *ymcA* deletion (green), and *ymcA* complemented strains (yellow and orange) to a severe heat shock at 54°C. Values were normalized to the viability at timepoint 0 min. The data represent the average ± standard error of three biological replicates. **D)** Growth curves of WT, Δ*ymcA*, and *ymcA* complemented strains in LB at 37°C. Three biological replicates are displayed with a smooth curve regression (LOESS) and a band representing the 95% confidence interval.

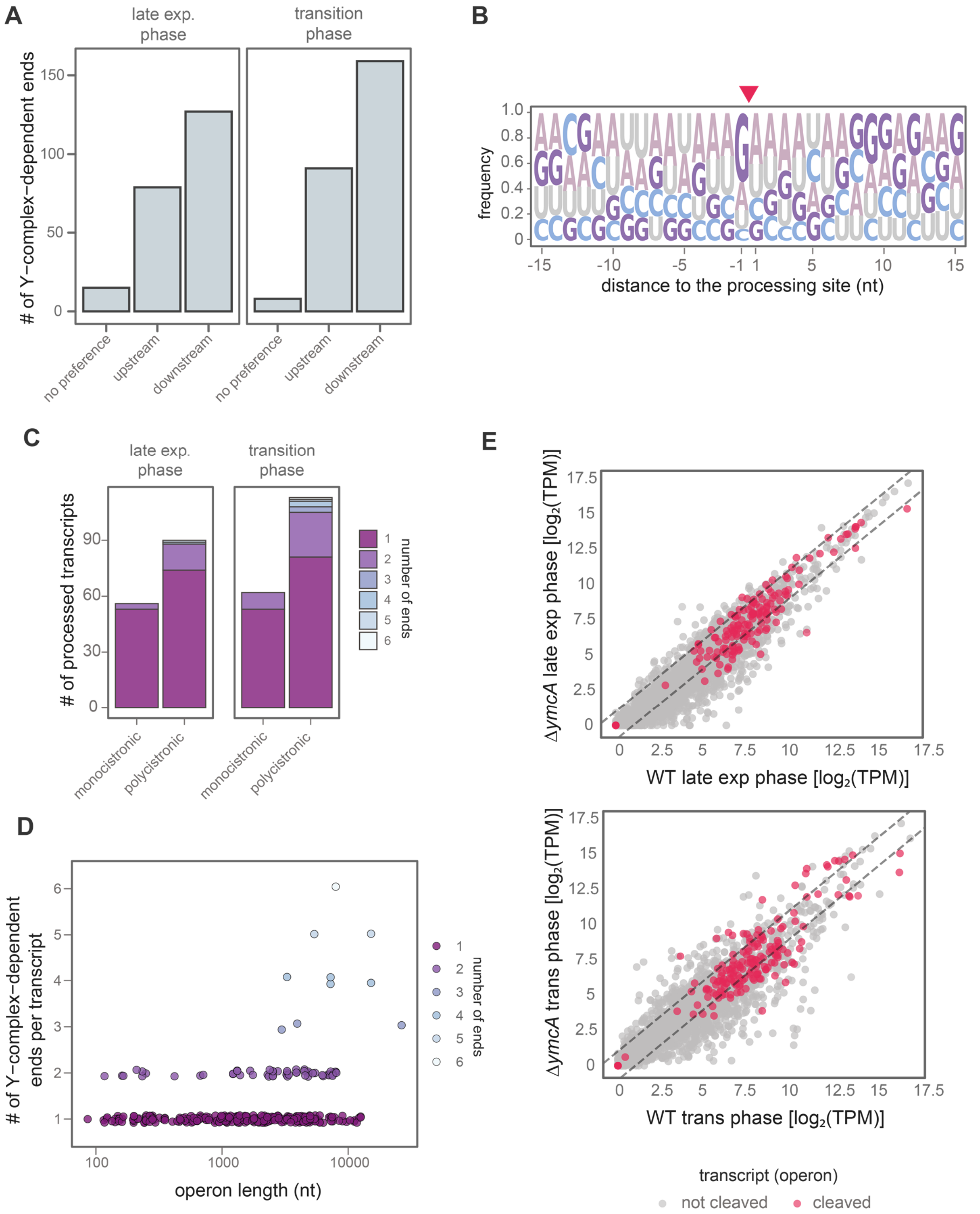

Supplementary Figure 2. Recognition motif, length, or expression levels do not determine the Y-complex substrates. A) Stabilization of the resulting fragments after Y-complex processing, the coverage ratio in a window of 5 nt before and after the processing site was calculated and used as a proxy of the stabilization of the resulting fragments. The predicted stability correlates with the end detected in the ISCP method. **B)** Motif search using 5' and U-type ends. The arrow indicates the cleavage site. **C)** Number of Y-complex-dependent cleavages detected per monocistronic or polycistronic transcripts in the late exponential or transition phase. **D)** Number of cleavages detected by operon length of the transcript. The color indicates the number of Y-complex-dependent ends detected per transcript **E)** RNA levels of WT and Δ*ymcA* mutant transcripts at late exponential and transition phases. Transcripts processed by the Y-complex are highlighted in magenta. Transcript annotations were obtained from BSGatlas.

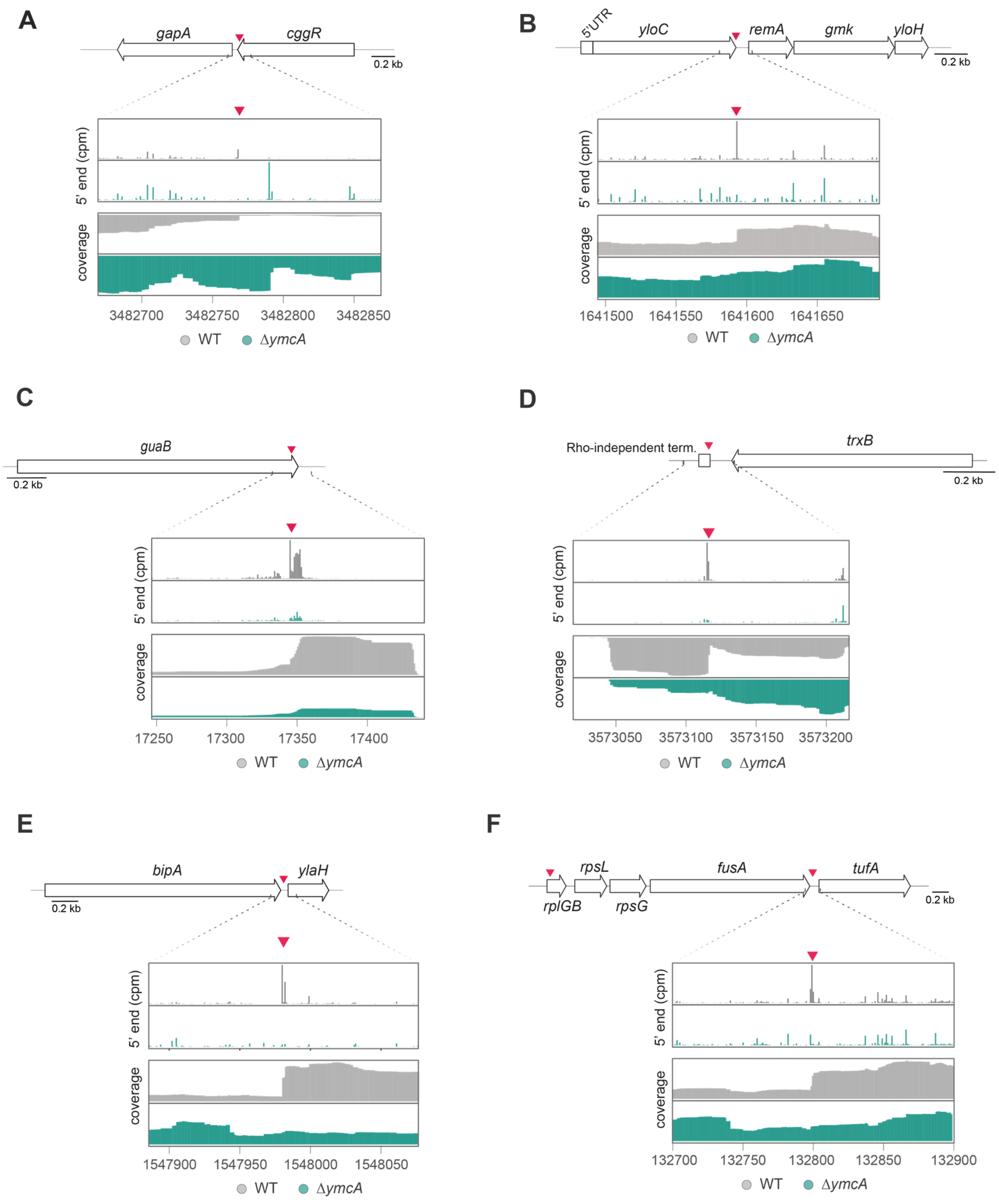

Supplementary Figure 3. The Y-complex processes mRNA in UTRs and coding regions. RNA end counts per million (cpm), and RNA-seq coverage of selected transcripts in WT (grey) and Δ*ymcA* (green). The arrow indicates the Y-complex-dependent processing site. The data shows the average of three biological replicates. **A)** The *gapA-cggR* transcript was used as a positive control. **B)** Processing in the coding region of *yloC* in the polycistronic mRNA *yloC-remA-gmk-yloH*. **C)** Processing in the coding region of the monocistronic transcript of *guaB*. **D)** Processing at the 3' UTR of *trxB,* near the Rho-independent terminator. Processing in the internal UTR of translation-associated transcripts like **E)** the *bipA-ylaH* transcript, and **F)** between *fusA-tufA*.

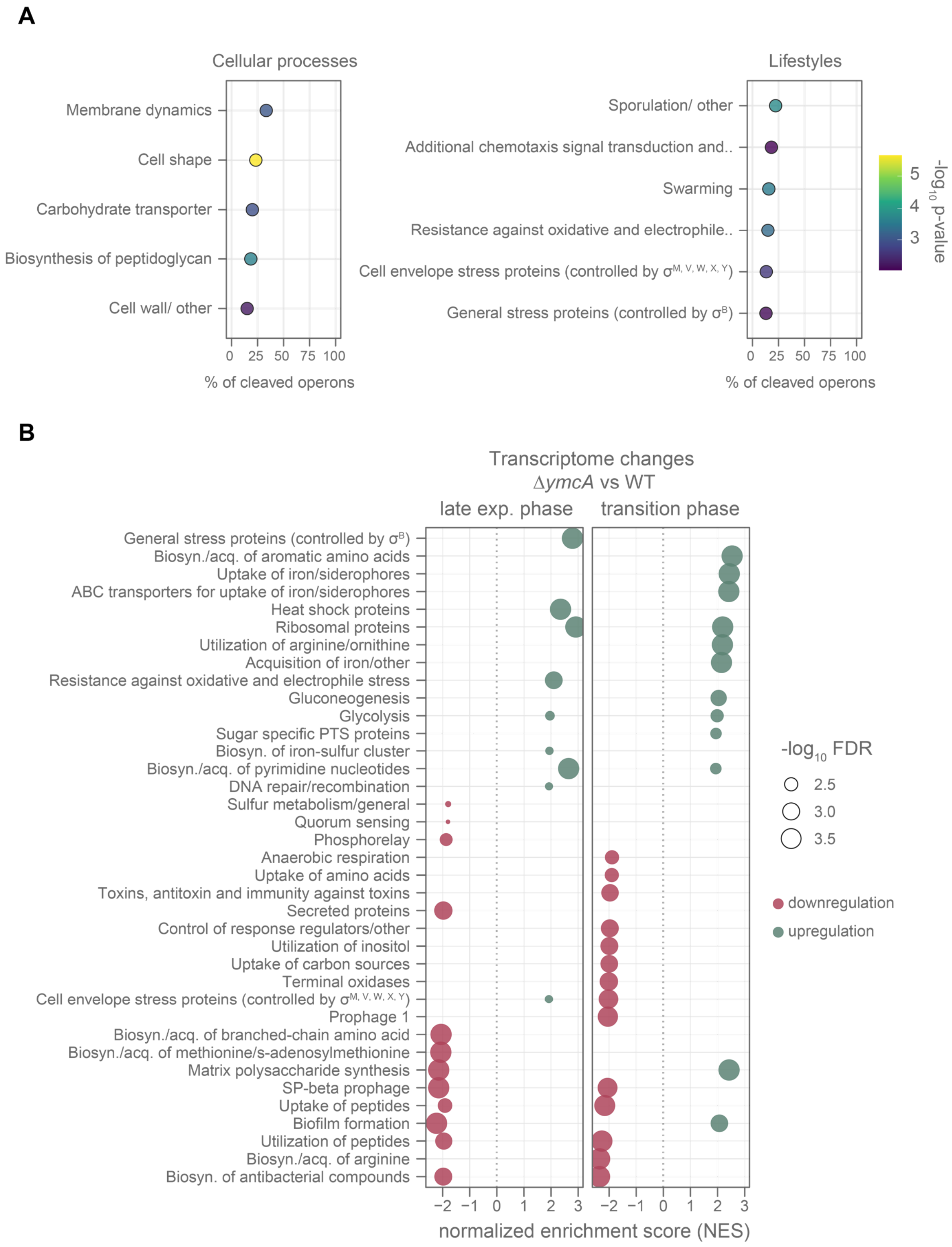

Supplementary Figure 4. mRNA processing by the Y-complex and effect on the transcriptome. A) Overrepresentation analysis of pathways whose transcripts are cleaved by the Y-complex during the late exponential phase. Operons were assigned to be involved in a pathway if they code for one gene participating in such a pathway. Pathway annotations were retrieved from SubtiWiki[48]. The color indicates the statistical significance of the enrichment. **B)** Gene set enrichment analysis (GSEA) of differentially expressed genes at the late exponential and transition phase when comparing Δ*ymcA* to WT. The size of the dot represents the statistical significance. The normalized enrichment score is displayed on the *x*-axis; positive values mean upregulation; meanwhile negative values mean

downregulation. GSEA results were prefiltered for pathways showing and enrichment with an FDR ≤ 0.01. Abbreviations: Biosyn. = Biosynthesis, acq. = Acquisition. Statistical significance and adjustment for multiple testing were performed using GSEA.

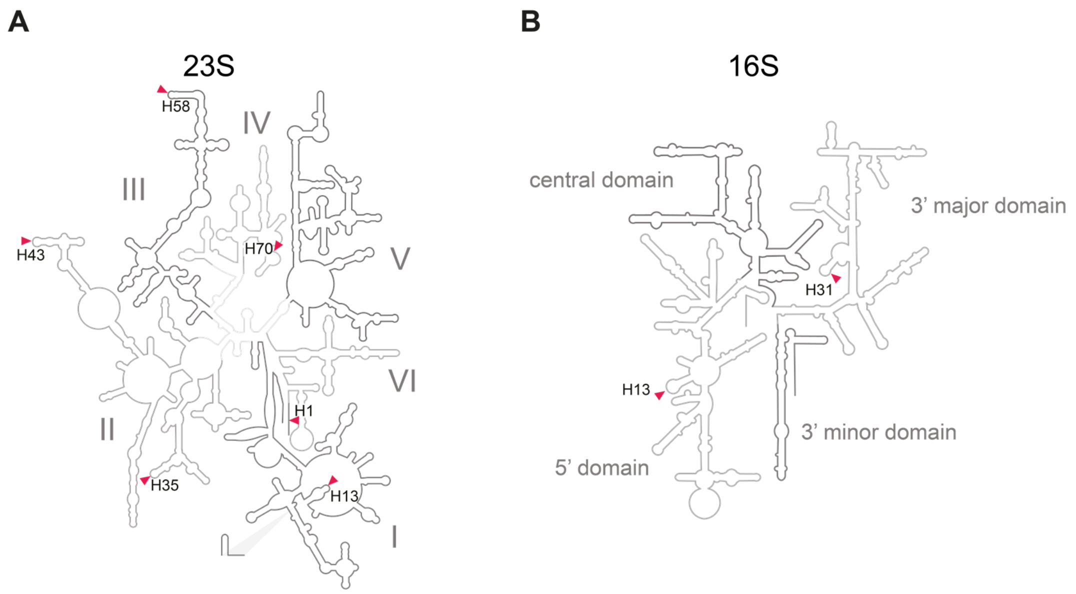

Supplementary Figure 5. Mapping of Y-complex-dependent processing in the secondary structure of rRNA at the late exponential phase. Cleavages are marked with an arrow and the helix number for **A)** 23S and **B)** 16S rRNA.

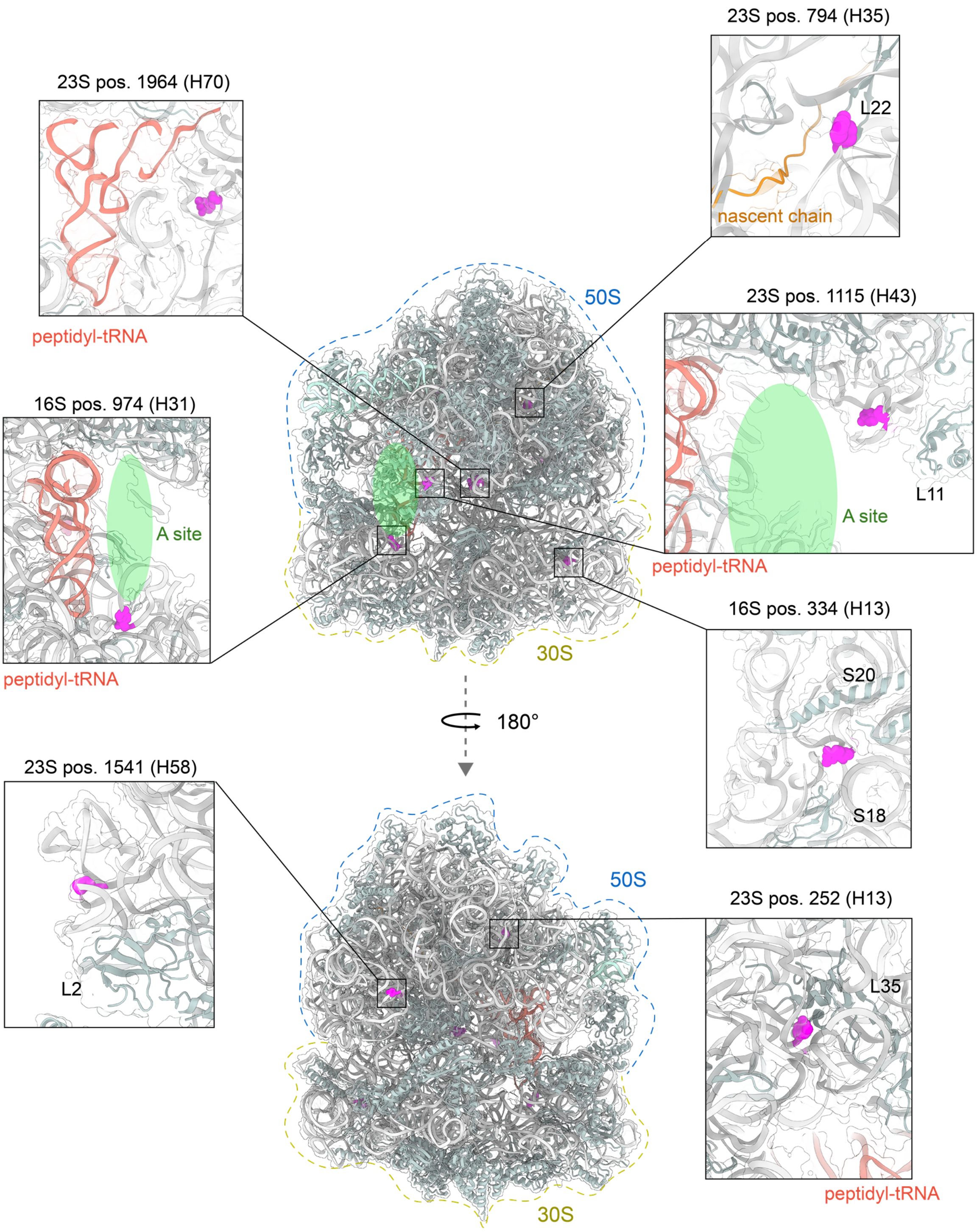

Supplementary Figure 6. Mapping of Y-complex-dependent processing in the ribosome 3D structure. The RNA end detected by the ISCP method is highlighted in magenta. The ribosome structure was retrieved from PDB (ID: 3J9W)[97,98]. Image created using ChimeraX[99].The A site is shown as a green ellipse.

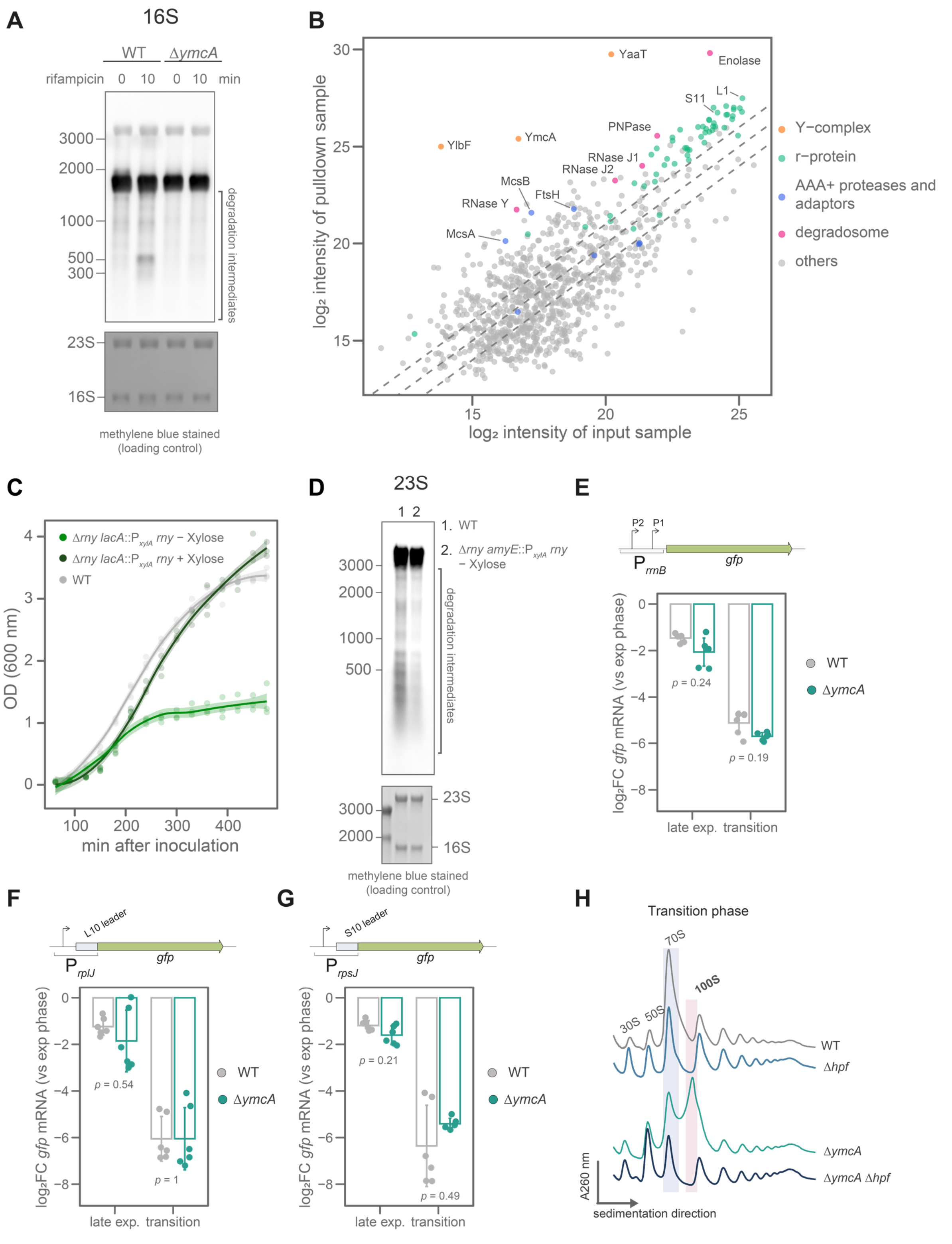

Supplementary Figure 7. The Y-complex and RNase Y initiate degradation of rRNA but does not affect transcriptional control of stringent response. A) Northern blot against 16S of WT and Δ*ymcA* cells at the late exponential phase, before and after treatment with 100 µg/ml rifampicin to inhibit transcription. The methylene blue-stained membrane is shown as a loading control. This image is representative of three biological replicates. **B)** Pulldown of YaaT-FLAG at the late exponential phase. The protein intensities of the input sample and the elution fraction were measured by mass spectrometry. The dotted lines indicate a fold change of less than 1. The data represent the mean of 3 replicates. **C)** Growth curve of RNase Y conditional depletion strains in LB. Expression of RNase Y

depends on the addition of xylose to the media. Three biological replicates are displayed with a smooth curve regression (LOESS) and a band representing the 95% confidence interval. **D)** Northern blot against 23S of WT and RNase Y depletion strains at the late exponential phase. The methylene blue-stained membrane is shown as a loading control. This image is representative of three biological replicates. Activity of the promoter controlling **E)** *rrnB* (rRNA) transcription, **F)** *rplJ*, and **G)** *rpsJ* (r-proteins) genes at late exponential and transition phase compared to the exponential phase in WT and Δ*ymcA*. The promoters were cloned controlling *gfp* transcription, which was measured using RT-qPCR. The *pcp* transcript was used as a reference. Individual values and the average ± standard deviation of three biological replicates and two technical replicates are shown. Statistical significance was assessed with a two-sided Student's *t*-test using the mean of technical replicates for each biological replicate. **H)** Ribosome sedimentation profile in 10-50% sucrose gradients of WT, Δ*hpf,* Δ*ymcA,* and Δ*ymcA* Δ*hpf* in the transition phase. The 70S and 100S ribosomes are highlighted with a blue or red box, respectively. The data is representative of three biological replicates.

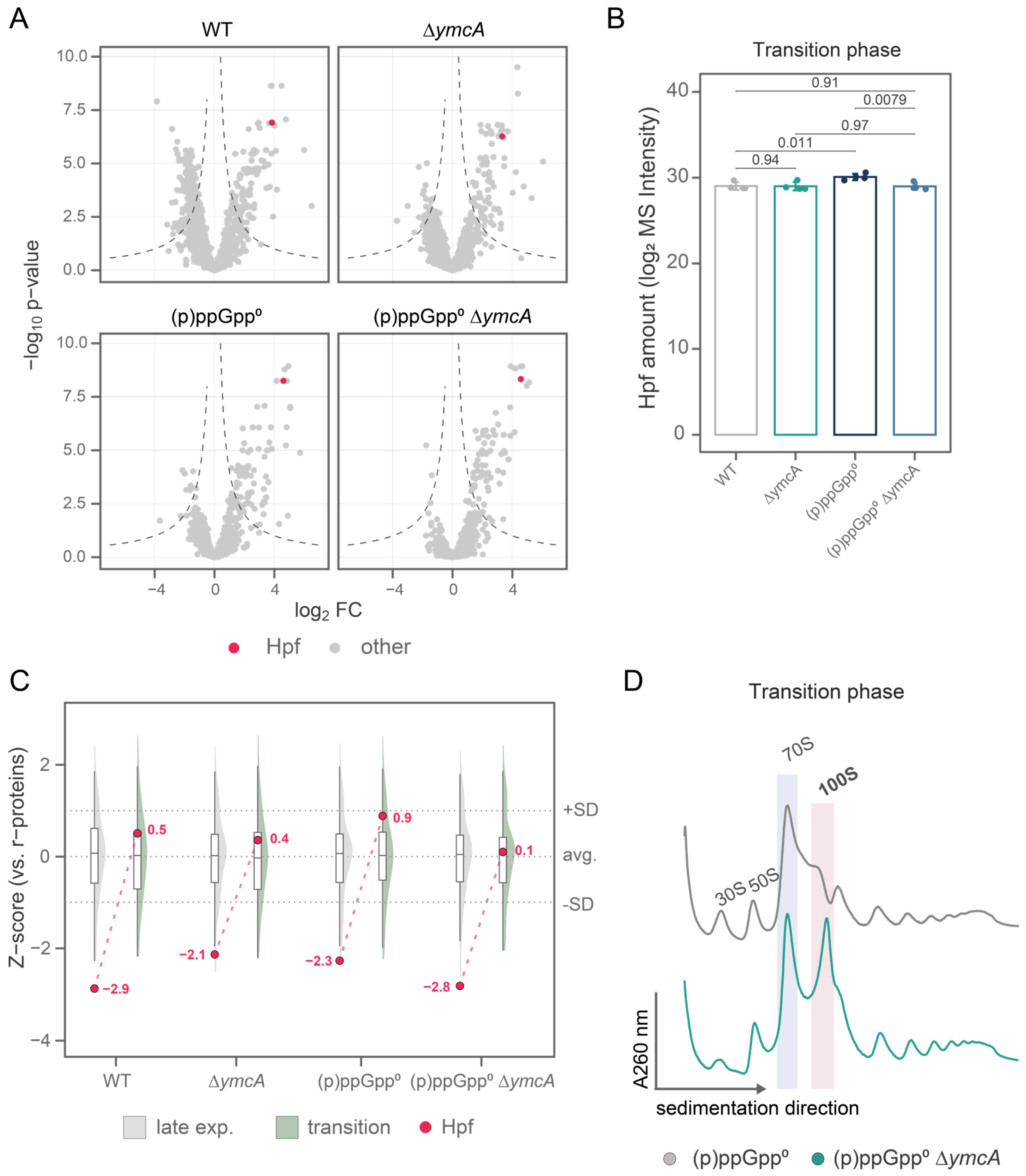

Supplementary Figure 8. 100S ribosome formation in (p)ppGpp0 strains suggests an additional alarmone-independent mechanism of ribosome hibernation. A) Volcano plot of proteomic changes of WT, Δ*ymcA,* (p)ppGpp^0, and (p)ppGpp^0 Δ*ymcA* cultures comparing transition to exponential phase. The Hpf protein is highlighted in magenta. The data shows the average of four biological replicates. The p-values were calculated using a two-sided Student's *t*-test and adjusted using the Benjamini-Hochberg method **B)** Hpf levels measured by mass spectrometry at the transition phase in the mentioned strains. The data shows the individual values of four biological replicates. Statistical significance (shown p-values) was assessed using a two-sided Student's *t*-test and adjusted for multiple testing using Holm's method. **C)** Hpf levels compared to r-proteins. The Z-score of r-proteins was calculated for each strain and time point independently. Hpf levels were Z-scored using the average and SD of the r-proteins. The boxplot represents the interquartile range (IQR) and the median in the center. Whiskers show the variability outside quartile 1 (Q1) and Q3 and were calculated as Q1-1.5*IQR and Q5+1.5*IQR, respectively. The average of four biological replicates was used for the analysis. **D)** Ribosome

sedimentation profile in 10-50% sucrose gradients of (p)ppGpp^0 and (p)ppGpp^0 Δ*ymcA* strains in the transition phase. The 70S and 100S ribosomes are highlighted with a blue or red box, respectively.

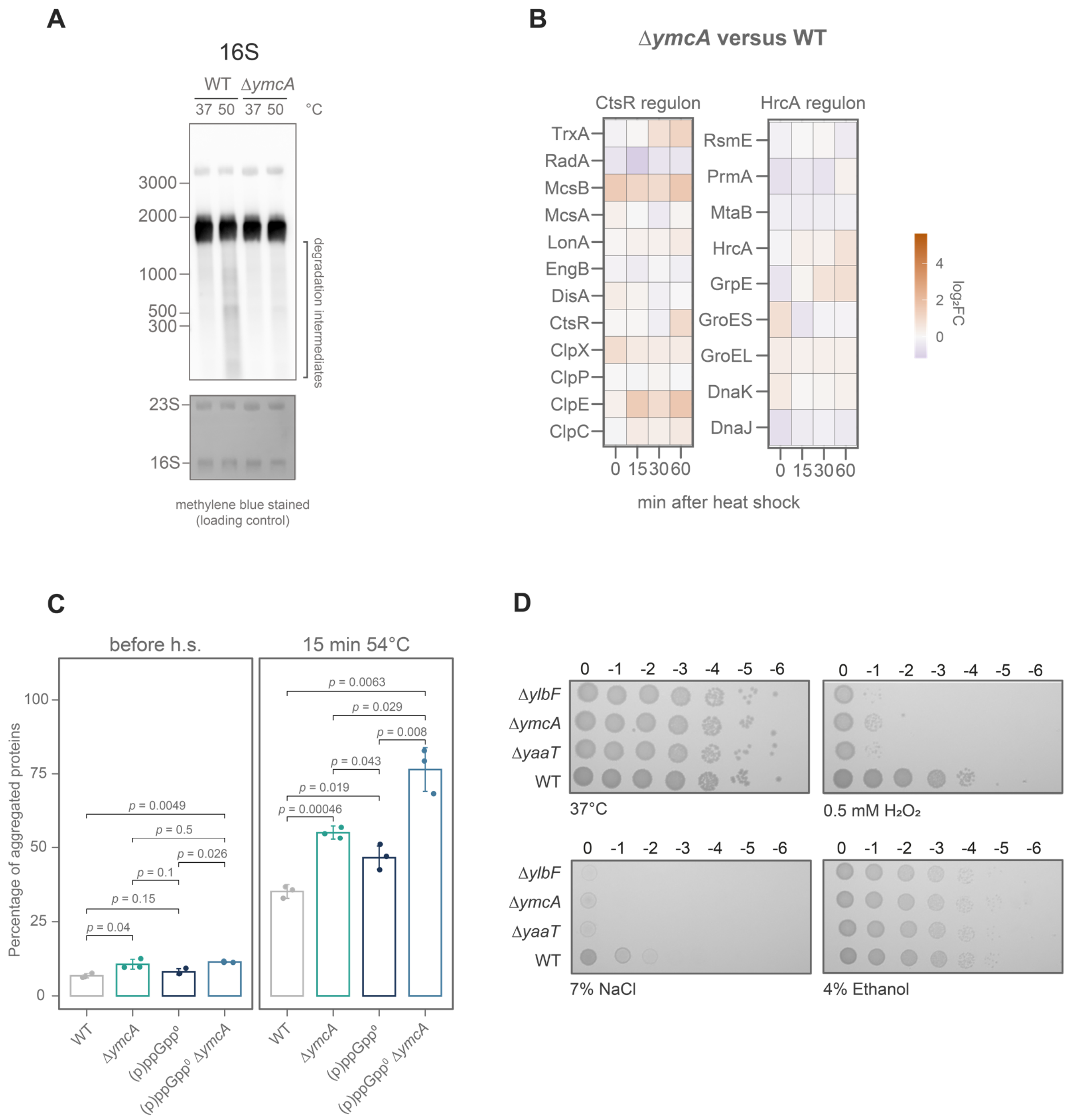

Supplementary Figure 9. Control of ribosome levels and protein homeostasis in *ymcA* is important for responding against proteotoxic stresses. A) Northern blot against 16S of WT and Δ*ymcA* cells at the exponential phase before and after heat shock. The methylene blue-stained membrane is shown as a loading control. This image is representative of three biological replicates. **B)** Induction of CtsR and HrcA regulons after heat shock (50°C) Δ*ymcA* compared to the WT. Proteins were measured by mass spectrometry. The color bar shows the $\log_2$ fold change compared to the WT control. **C)** Percentage of aggregated proteins before and after a 15 min heat shock at 54°C in WT and Δ*ymcA*, (p)ppGpp^0 and (p)ppGpp^0 Δ*ymcA*. Individual values and the average ± standard deviation of three biological replicates are shown. Statistical significance (p-value) was assessed using a two-sided Student's *t*-test and adjusted for multiple testing using Holm's method. **D)** Spot test of deletion mutants of Y-complex members in the displayed stresses. The picture is representative of three biological replicates.
