# Supplementary material for: Stress-induced ribosome degradation in Bacillus subtilis is mediated by the RNase Y-specificity complex
Source: Nat Commun. 2026 Jun 2;17:4886. doi: 10.1038/s41467-026-73310-x (PMC13230545; doi:10.1038/s41467-026-73310-x)
Supplement: Supplementary file 2 — Description of Additional Supplementary Files [file 41467_2026_73310_MOESM2_ESM.pdf]

Description of Additional Supplementary Files

File Name: Supplementary Data 1

Description: Fitness of deletion mutants after a heat shock (50°C) or a control kept at (30°C). Statistical significance (p-value) was tested using a two-sided Student's t-test and adjusted using the Benjamini-Hochberg method.

File Name: Supplementary Data 2

Description: Identified *ymcA*-dependent cleavages at both growth phases. Annotation was retrieved from BSGatlas. Statistical significance (p-value) was calculated using edgeR.

File Name: Supplementary Data 3

Description: Overlap between *ymcA*-dependent cleavages at late exponential and transition phase found in this study and described cleavages at mid-exponential phase.

File Name: Supplementary Data 4

Description: Expression levels of operons at late exp. and transition phase. Transcripts per million (TPM) of operons in WT and Δ*ymcA* at the two growth phases. Information if a cleavage was detected in the respective operon is provided. Operon annotation was retrieved from BSGatlas.

File Name: Supplementary Data 5

Description: Overrepresentation analysis of cleaved transcripts. Gene categories annotation was retrieved from SubtiWiki. p-values were calculated using a one-sided hypergeometric test and adjusted using the Benjamini-Hochberg method

File Name: Supplementary Data 6

Description: Differential expression analysis (RNA-seq) of WT and Δ*ymcA* at late exponential and transition phase. Statistical differences (p-values) were calculated using DESeq2 and corrected for multiple testing using the Benjamini-Hochberg method.

File Name: Supplementary Data 7

Description: Gene set enrichment analysis of SubtiWiki gene categories affected by *ymcA* deletion. ES = Enrichment score, NES = Normalized enrichment score. Statistical significance (p-value) and multiple testing adjustment were performed using GSEA.

File Name: Supplementary Data 8

Description: Proteins identified in YaaT-FLAG pulldown by DIA-MS. Proteins with a delta of intensities equal to or greater than 1 were considered enriched by the pulldown. Categories are highlighted as in the plot in Supplementary Figure 7B.

File Name: Supplementary Data 9

Description: Proteome changes of WT, Δ*ymcA*, (p)ppGpp^0 and (p)ppGpp^0 Δ*ymcA* at transition phase compared to the late exponential phase. Statistical significance (p-values; two-sided t-test) and multiple testing adjustment (Benjamini-Hochberg) were performed using MSstats.

File Name: Supplementary Data 10

Description: Proteome changes of WT and Δ*ymcA* during a heat shock at 50°C. Statistical significance (p-values; two-sided t-test) and multiple testing adjustment (Benjamini-Hochberg) were performed using MSstats.

File Name: Supplementary Data 11

Description: Strains used in this study.

File Name: Supplementary Data 12

Description: Primers used for library sequencing, qPCR, and synthesis of Northern blot probes.
