# Supplementary material for: Stress-induced ribosome degradation in Bacillus subtilis is mediated by the RNase Y-specificity complex
Source: Nat Commun. 2026 Jun 2;17:4886. doi: 10.1038/s41467-026-73310-x (PMC13230545; doi:10.1038/s41467-026-73310-x)
Supplement: Supplementary file 4 — Reporting Summary [file 41467_2026_73310_MOESM4_ESM.pdf]

nature portfolio

Corresponding author(s): Fabián Cornejo, Kürsad Turgay

Last updated by author(s): Apr 29, 2026

Reporting Summary

Nature Portfolio wishes to improve the reproducibility of the work that we publish. This form provides structure for consistency and transparency in reporting. For further information on Nature Portfolio policies, see our Editorial Policies and the Editorial Policy Checklist.

Statistics

For all statistical analyses, confirm that the following items are present in the figure legend, table legend, main text, or Methods section.

n/a	Confirmed	
☐	☒	The exact sample size (*n*) for each experimental group/condition, given as a discrete number and unit of measurement
☐	☒	A statement on whether measurements were taken from distinct samples or whether the same sample was measured repeatedly
☐	☒	The statistical test(s) used AND whether they are one- or two-sided<br>*Only common tests should be described solely by name; describe more complex techniques in the Methods section.*
☒	☐	A description of all covariates tested
☐	☒	A description of any assumptions or corrections, such as tests of normality and adjustment for multiple comparisons
☐	☒	A full description of the statistical parameters including central tendency (e.g. means) or other basic estimates (e.g. regression coefficient) AND variation (e.g. standard deviation) or associated estimates of uncertainty (e.g. confidence intervals)
☐	☒	For null hypothesis testing, the test statistic (e.g. *F*, *t*, *r*) with confidence intervals, effect sizes, degrees of freedom and *P* value noted<br>*Give P values as exact values whenever suitable.*
☒	☐	For Bayesian analysis, information on the choice of priors and Markov chain Monte Carlo settings
☒	☐	For hierarchical and complex designs, identification of the appropriate level for tests and full reporting of outcomes
☒	☐	Estimates of effect sizes (e.g. Cohen's *d*, Pearson's *r*), indicating how they were calculated

Our web collection on statistics for biologists contains articles on many of the points above.

Software and code

Policy information about availability of computer code

Data collection	For plate reader measurement (incl. OD): Gen5 v. 3.10 & 3.12, Northern blot and plate pictures: Evolution Capt Edge, RT-qPCR: QuantStudio Design & Analysis Software v.1.5.3, Sucrose gradients: BioComp FlowCell Software, Mass spectrometry: Orbitrap Exploris 480 Tune Application: v. 4.2.362.42 & Orbitrap Fusion Lumos Tune Application: v. 4.1.4244, RNA-seq: Sequencing platforms NextSeq - NovaSeq. For Microscopy: LASX v. 3.7.6.25997.
Data analysis	For processing RNA sequencing data we utilized: FastQC (v0.11.5), Cutadapt (v3.5), STAR (v2.7.10a_alpha_220314), Samtools (v1.9), UMI Tools (v1.1.0), featureCounts (v2.0.3), DESeq2 (v1.24.0), HTSeq library (v2.0.2) implemented in Snakemake (v7.14.0)<br>For identifying and analyzing Y-complex-dependent cleavage sites: We used the procedure described in Lécrivain et al. 2018 (https://doi.org/10.1073/pnas.1809663115), edgeR (v3.28.0), Logomaker (v0.8), RNAfold (v2.6.4) and and the Bioconductor package Category (v.2.64.0) in R (v.4.2.2). In addition, biopython (v. 1.85) and Python (v. 3) were used.<br>For proteomic measurements: we utilized MSFragger (v.3.5), (DIA-NN v1.8.1) for DIA data analysis and MSFragger for DDA data analysis.<br>The Bioconductor package MSstats (v.4.12.1) was used for downstream analysis.<br>Enrichment analysis were performed using GSEA (v. 4.1)<br>Microscopy analysis was performed using Fiji (ImageJ v. 1.54f) and MicrobeJ (v. 5.13p)<br>For plotting and performing some of the statistical analyses we used different versions over the years of the R packages tidyverse (v 2.0), ggpubr (v 0.6.1) and ggstatsplot (v 0.13.6)<br>Structure visualization was performed with ChimeraX (v. 1.9)

For manuscripts utilizing custom algorithms or software that are central to the research but not yet described in published literature, software must be made available to editors and reviewers. We strongly encourage code deposition in a community repository (e.g. GitHub). See the Nature Portfolio guidelines for submitting code & software for further information.

Data

Policy information about availability of data

All manuscripts must include a data availability statement. This statement should provide the following information, where applicable:

- Accession codes, unique identifiers, or web links for publicly available datasets
- A description of any restrictions on data availability
- For clinical datasets or third party data, please ensure that the statement adheres to our policy

The Amplicon-seq and RNA-seq raw reads generated in this study have been deposited at the European Nucleotide Archive (ENA) under accession number PRJEB90661 [https://www.ebi.ac.uk/ena/browser/view/PRJEB90661]. Raw proteomic data have been deposited at the PRIDE database under the accession number PXD065465 [https://www.ebi.ac.uk/pride/archive/projects/PXD065465] for DDA proteomics of late exponential and transition phase samples. The DIA proteomics raw data from the heat shock experiment have been deposited at the PRIDE database under the accession number PXD065462 [https://www.ebi.ac.uk/pride/archive/projects/PXD065462]. The YaaT-pulldown proteomic data are accessible using the accession number PXD074523 [https://www.ebi.ac.uk/pride/archive/projects/PXD074523] in PRIDE. The Source data for each figure are provided with this paper. It can also be found in EDMOND [https://doi.org/10.17617/3.E9LOGJ], along with raw microscopy images, processed sequencing data and the code for Figure 2D, Supplementary Figure 2B and Supplementary Figure 3.

Research involving human participants, their data, or biological material

Policy information about studies with human participants or human data. See also policy information about sex, gender (identity/presentation), and sexual orientation and race, ethnicity and racism.

Reporting on sex and gender	No human participants, biological material or samples were used in this study
Reporting on race, ethnicity, or other socially relevant groupings	No human participants, biological material or samples were used in this study
Population characteristics	No human participants, biological material or samples were used in this study
Recruitment	No human participants, biological material or samples were used in this study
Ethics oversight	No human participants, biological material or samples were used in this study

Note that full information on the approval of the study protocol must also be provided in the manuscript.

Field-specific reporting

Please select the one below that is the best fit for your research. If you are not sure, read the appropriate sections before making your selection.

☒ Life sciences ☐ Behavioural & social sciences ☐ Ecological, evolutionary & environmental sciences

For a reference copy of the document with all sections, see nature.com/documents/nr-reporting-summary-flat.pdf

Life sciences study design

All studies must disclose on these points even when the disclosure is negative.

Sample size	Sample-size calculations were not conducted. The number of replicates in each experiment was adequate for hypothesis testing.
Data exclusions	In Figure 4C, alternative stress-induced r-proteins were removed for visualization reasons. Their expression changes can still be visualized in Figures 3C and 4B.
Replication	Unless stated the contrary, experiments were performed in at least 3 biological replicates (this information can be found in the figure legends). For RT-qPCR measurements we also included technical duplicates in addition to the three biological replicates. Microscopy-based half life determinations were performed in two independent experiments, but over one hundred cells were quantified for each replicate, time point and strain.
Randomization	No randomization was necessary for this study
Blinding	Blinding was not relevant for our study. We performed computational analysis that were insensitive to human bias.

Reporting for specific materials, systems and methods

We require information from authors about some types of materials, experimental systems and methods used in many studies. Here, indicate whether each material, system or method listed is relevant to your study. If you are not sure if a list item applies to your research, read the appropriate section before selecting a response.

Materials & experimental systems

n/a	Involved in the study
☒	☐ Antibodies
☒	☐ Eukaryotic cell lines
☒	☐ Palaeontology and archaeology
☒	☐ Animals and other organisms
☒	☐ Clinical data
☒	☐ Dual use research of concern
☒	☐ Plants

Methods

n/a	Involved in the study
☒	☐ ChIP-seq
☒	☐ Flow cytometry
☒	☐ MRI-based neuroimaging

Plants

Seed stocks	No plants samples were used in this study
Novel plant genotypes	No plants samples were used in this study
Authentication	No plants samples were used in this study
